# The bHLH transcription factor *AhbHLH112* improves the drought tolerance of peanut

**DOI:** 10.1186/s12870-021-03318-6

**Published:** 2021-11-16

**Authors:** Chunjuan Li, Caixia Yan, Quanxi Sun, Juan Wang, Cuiling Yuan, Yifei Mou, Shihua Shan, Xiaobo Zhao

**Affiliations:** Department of Breeding, Shandong Peanut Research Institute, Qingdao, China

**Keywords:** Basic helix–loop–helix transcription factors, Peanut, Drought stress, Transcriptional regulation, ROS homeostasis

## Abstract

**Background:**

Basic helix-loop-helix (bHLH) transcription factors (TFs) are one of the largest gene families in plants. They regulate gene expression through interactions with specific motifs in target genes. bHLH TFs are not only universally involved in plant growth but also play an important role in plant responses to abiotic stress. However, most members of this family have not been functionally characterized.

**Results:**

Here, we characterized the function of a bHLH TF in the peanut, *AhHLH112*, in response to drought stress. AhHLH112 is localized in the nucleus and it was induced by drought stress. The overexpression of this gene improves the drought tolerance of transgenic plants both in seedling and adult stages. Compared to wild-type plants, the transgenic plants accumulated less reactive oxygen species (ROS), accompanied by increased activity and transcript levels of antioxidant enzymes (superoxide dismutase, peroxidase and catalase). In addition, the WT plants demonstrated higher MDA concentration levels and higher water loss rate than the transgenic plants under drought treatment. The Yeast one-hybrid result also demonstrates that AhbHLH112 directly and specifically binds to and activates the promoter of the *peroxidase* (POD) gene. Besides, overexpression of *AhHLH112* improved ABA level under drought condition, and elevated the expression of genes associated with ABA biosynthesis and ABA responding, including *AtNCED3* and *AtRD29A*.

**Conclusions:**

Drawing on the results of our experiments, we propose that, by improving ROS-scavenging ability, at least in part through the regulation of POD -mediated H_2_O_2_ homeostasis, and possibly participates in ABA-dependent stress-responding pathway, *AhbHLH112* acts as a positive factor in drought stress tolerance.

**Supplementary Information:**

The online version contains supplementary material available at 10.1186/s12870-021-03318-6.

## Background

The peanut (*Arachis hypogaea* L.) is an important food source for nutritious oil and protein and is cultivated in semiarid tropical and subtropical regions worldwide [1]. The cultivated peanut is an allotetraploid (AABB-type genome: 2n = 4x = 40), probably derived from a single recent hybridization event between two diploid wild species (*Arachis duranensis* [AA-type genome; 2n = 2x = 20] and *Arachis ipaensis* [BB-type genome; 2n = 2x = 20]) through polyploidization, followed by subsequent spontaneous genome duplication [2, 3]. *Arachis* allotetraploids are larger than their diploid progenitors. The tetraploids also have different transpiration characteristics and produce more photosynthetic pigments. These changes may have been advantageous; however, the increased number of alleles associated with being a ‘fixed hybrid’ would have increased heterosis and therefore probably adaptability [3]. Similar advantage is also observed in other plants, such as cotton (*Gossypium*). The allotetraploid cotton, currently dominates the world’s cotton commerce for its higher yield and superior fiber quality, comparing with the diploid species [4].

Although plants adopt defense mechanisms, including morphological [5], physiological [6], and molecular mechanisms [7], to cope with drought, it remains one of the main factors that limit the growth of peanuts [8]. Therefore, a major future challenge is how to sustain and even increase peanut production, even while conditions are deteriorating, to meet growing needs. To achieve this goal, a number of drought-resistant genes must be identified. Unfortunately, studies of drought-resistant genes in the peanut are rare, compared with those of other plants.

Transcription factors (TFs) are important regulatory proteins that function to control the expression of target genes. Therefore, the identification and characterization of drought-responsive TFs is crucial for elucidating the molecular network associated with drought response [9]. Basic helix-loop-helix (bHLH) TFs are among the largest groups of TFs and in animals, yeasts, and plants [10]. They are generally found at the N-terminus of the conserved bHLH domain and consist of approximately 15 to 20 residues, which function together as a DNA-binding motif [11, 12]. The HLH domain of bHLH TFs is at the C-terminus of the amino acid sequence and is composed of two amphipathic α-helices mainly incorporating hydrophobic residues linked by a loop region of a variable sequence and length [13]. The first member of this family was discovered in maize and interacts with members of the MYB family to control anthocyanin biosynthesis and pigmentation [14]. In all, 167, 165, 159, 127, 85, 107 and 97 bHLH family members have been found in *Arabidopsis* [15], *Oryza* [16], *Solanum* [17], *Salvia miltiorrhiza* [18], *Ginkgo biloba* [19], *Capsicum* [20] and *Carica papaya* [21], respectively. Genome-wide analyses have identified 261 *bHLH* genes in the peanut [22]. In addition, increasing numbers of *bHLH* gens are being identified in plants.

In plants, *bHLH* genes are involved in drought tolerance. *MdbHLH130* from apple improves water-deficit stress tolerance in transgenic tobacco by maintaining the homeostasis of reactive oxygen species (ROS) [23]. The transgenic expression of *PebHLH35* from the desert poplar (*Populus euphratica* O.) in *Arabidopsis* increases tolerance to water deficit stress by regulating stomatal development and photosynthesis in the resulting plants [24]. The bHLH-induced enhancement of plant drought tolerance is also related to abscisic acid (ABA) signaling. In wheat, drought adaptability is improved by the regulation of the ABA pathway by the *TabHLH1* gene [25]. In rice, the over-expression of *OsbHLH148*, which regulates the JA pathway and the function of the jasmonate ZIM domain (OsJAZ) protein, increases drought tolerance of the plants [26]. Unfortunately, studies on *bHLH* genes in peanut are rare, and most have focused on their roles in development [27].

Here, we focused on *AhbHLH112*, one of the most drought-inducible TFs in previous research. Our goal was to verify the roles of this TF in improving drought tolerance and investigate the underlying mechanisms. Our data provide a comprehensive resource for further molecular research on this species.

## Results

### Cloning and expression of AhbHLH112

In our previous transcriptomic analyse in the response of peanut to drought stress [1], we identified some *bHLH* genes as positive regulators, including *AhbHLH11*2. This study is our first step to research these TFs. *AhbHLH11*2 coding DNA sequence (CDS) is 1344 bp in length and encodes 447 amino acid residues, with a predicted molecular mass of 48.7 kDa and a theoretical isoelectric point of 4.54. Our result was accordance with the data obtained from PeanutBase (Arahy.0I3NZ2; www. peanutbase.org). However, in another genome database (http://peanutgr.fafu.edu.cn/index.php; AH10G04090), at site 951, C was instead of G in *AhbHLH11*2 (Fig. S1). A comparison of the genomic DNA and cDNA sequences revealed that this gene has six intron regions (Fig. S2). Conserved domain analyse indicated that AhbHLH112 protein contains a typical bHLH domain (amino acids 323–373) (Fig. S3). Phylogenetic tree analyses indicated that the AhbHLH112 protein was clustered within the same clade as AtbHLH112 (At1g61660) of *Arabidopsis* and belonged to the bHLH s subgroup 12 (Fig. S4).

**Fig. 1:**
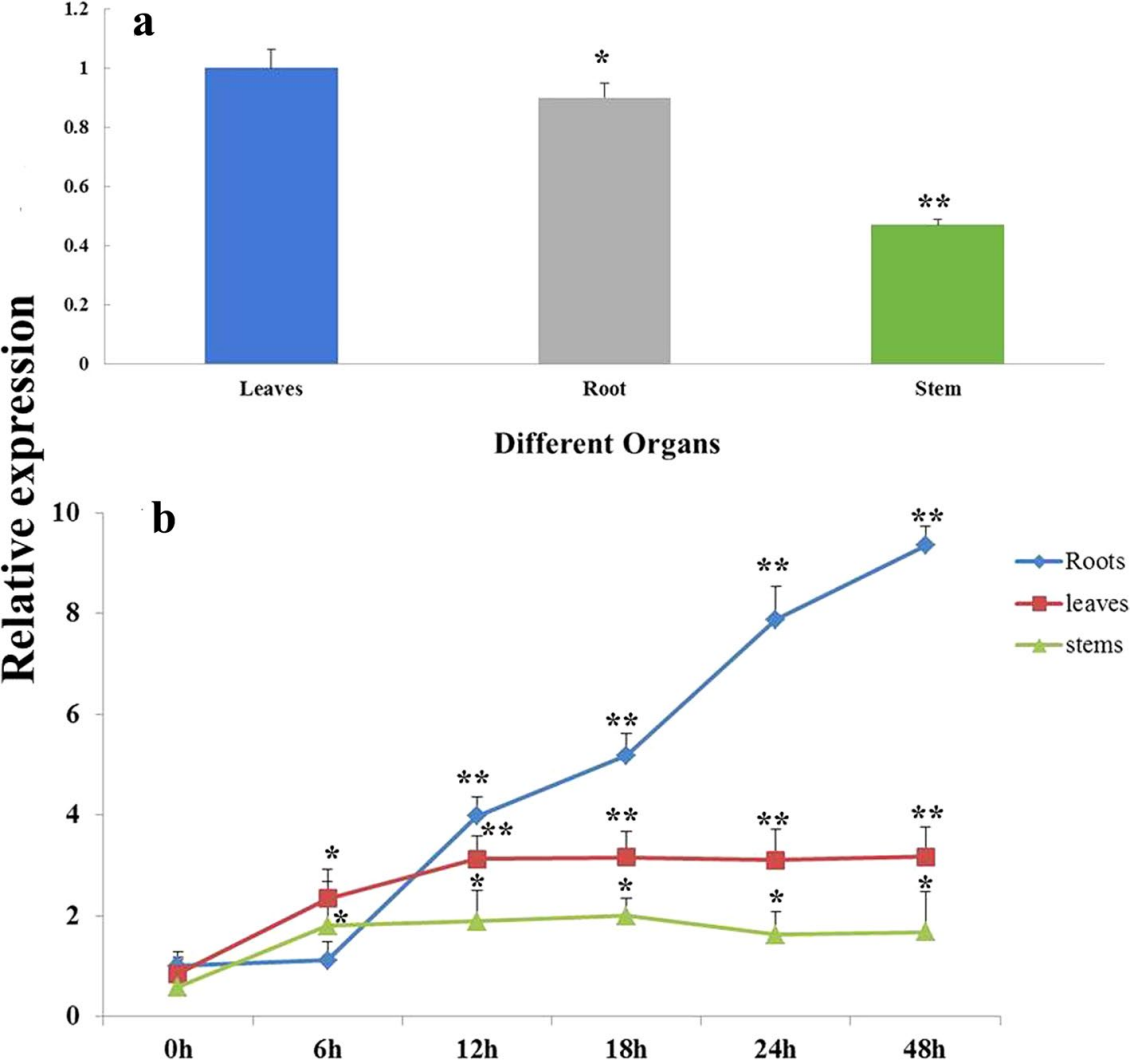
Expression analyse of *AhbHLH112* in different tissues under normal and drought stress conditions. *Actin11* was used as an internal reference control, and transcript levels of the tested genes were calculated using the 2 ^−∆∆CT^ method. Error bars represent SDs for three independent replicates. **a**: Detection of *AhbHLH11*2 transcript in different tissues of peanut plant under normal condition. Asterisks indicate difference (*P < 0.05) and significant difference (**P < 0.01) comparing to relative expression in leaves; (**b**): Expression of *AhbHLH11*2 in different tissues in response to drought. Asterisks indicate difference (*P < 0.05) and significant difference (**P < 0.01) comparing torelative expression at 0 h

The RT-qPCR results indicated that *AhbHLH112* was expressed in the roots, stems, and leaves of *A. hypogaea* under normal conditions, with the highest transcript levels in the leaves, followed by the roots (Fig. 1a). The data from our drought stress experiments showed that expression in all tissues dramatically increased in response to drought stress. In the leaves, the expression remained stable after 12 h. In the roots, the expression significantly increased within 48 h. In the stems, the expression gradually returned to its 6 h level after 18 h (Fig. 1b).

### Subcellular localization and transcriptional activity analyses of AhbHLH112

**Fig. 2:**
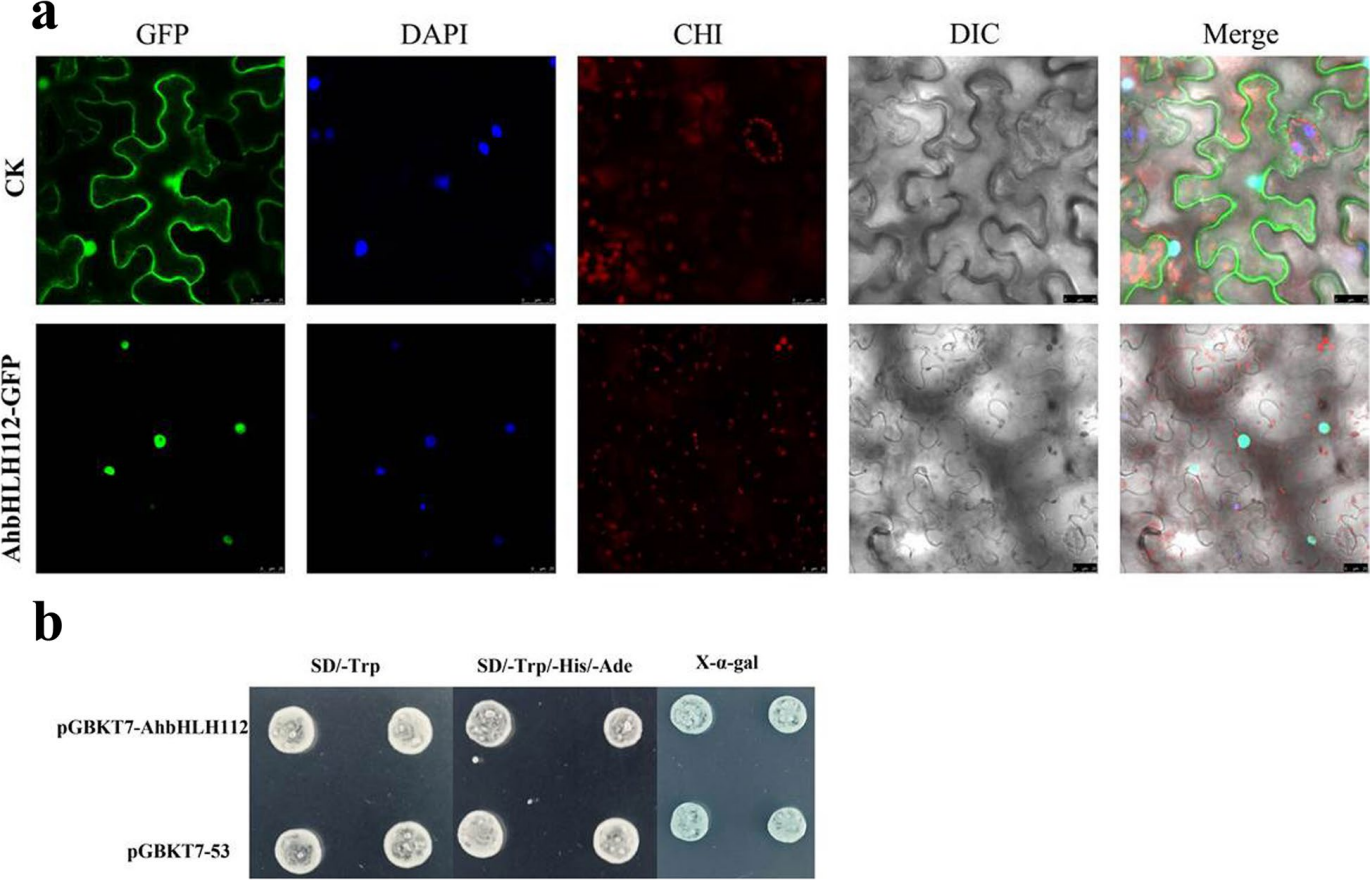
Subcellular localization and transcriptional activity of AhbHLH112. **a**: Subcellular localization of AhbHLH112 protein. CK: pCAMBIA2300-GFP; Bar = 25 μM. **b**: Growth and X-α-GAL staining assay of transformant colonies on SD/−Trp and SD/−Trp/−His/−Ade medium. The pGBKT7–53 vector was used as a positive control

The subcellular localization of proteins is helpful in functional analyses. Due to their putative role in transcriptional regulation, TFs are thought to localize to the nucleus. By examining the subcellular distribution of the AhbHLH112 protein, we determined that the fluorescence signal for our positive CK was strong throughout whole cells. For *AhbHLH112*, the fluorescence signal was found in the nucleus (Fig. 2a). Yeast cells transformed with pGBKT7-AhbHLH112 and pGBKT7–53 (CK) grew well on selection media, including SD/−Trp and SD/−Trp/−His/−Ade with β-galactosidase activity (Fig. 2b). Thus, *AhbHLH112* has transcriptional activity.

### Overexpression of *AhHLH112* improves the drought tolerance of transgenic *A. thaliana* plants

**Fig. 3:**
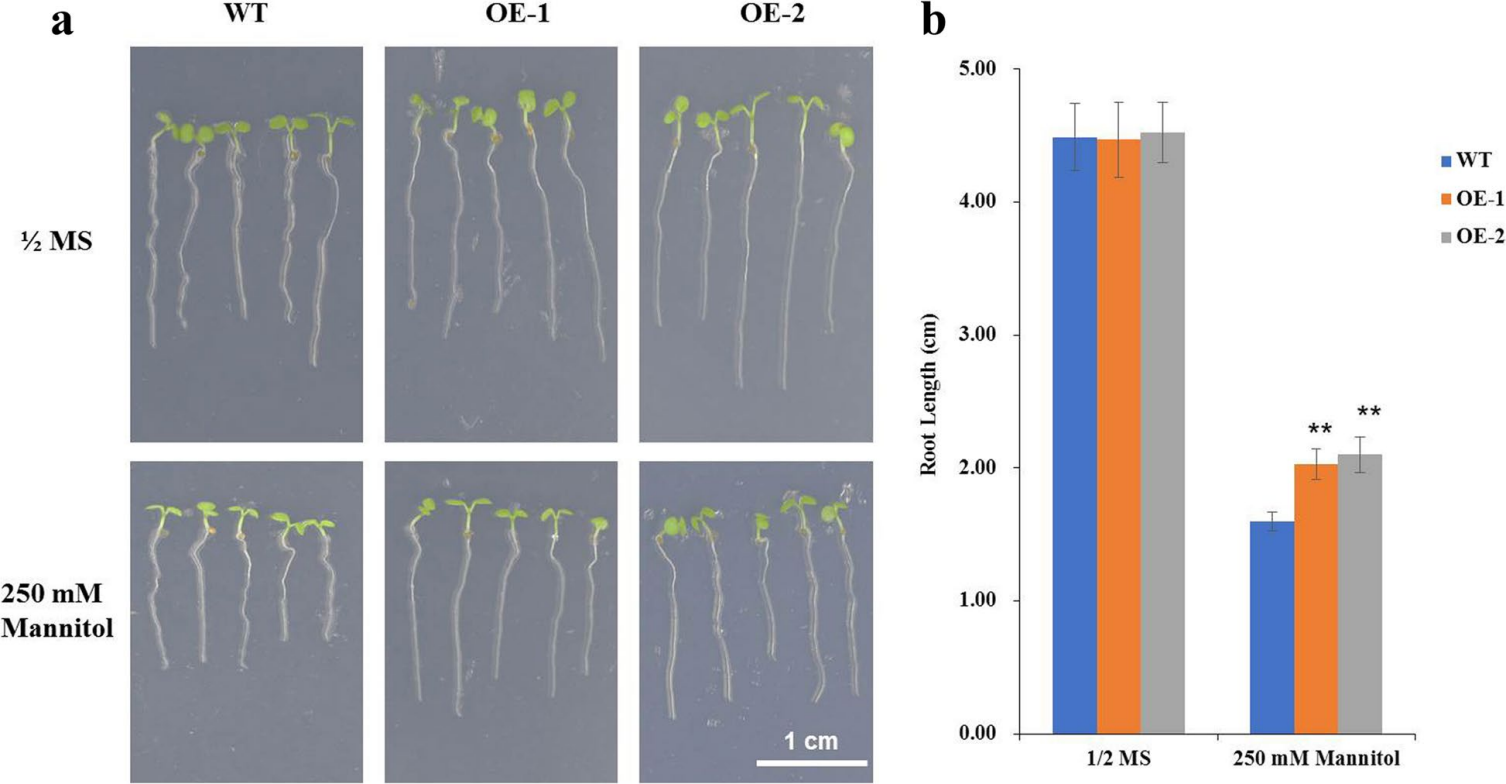
Analyse of drought a tolerance at the seedling stage. **a**: Morphology of transgenic and WT seedlings growing for nine days on 1/2 MS medium with mannitol. **b**: The analysis of root length of transgenic and WT plants. Y-ray is the root length (cm). Data are presented as means and SDs of three independent experiments. Asterisks indicate difference (*P < 0.05) and significant difference (**P < 0.01) comparing to WT.

To determine the roles of the *AhHLH112* TF in plants, its sequence was inserted into a pCAMBIA2300 vector and then overexpressed in *Arabidopsis*. Transformants were selected according to their kanamycin resistance and verified via PCR (Fig. S5). Afterward, *AhHLH112* expression in transgenic *Arabidopsis* was measured via RT-PCR. A single specific band was observed in each transgenic line, while no band was observed in the wild type (WT) CK (Fig. S6). To verify whether *AhHLH112* was associated with drought stress tolerance, the WT and transgenic lines were subjected to stress treatments at both the seedling and adult stages. In the seedling stage, there were no significant differences between the WT plants and transgenic plants under normal conditions. Under mannitol treatment, the transgenic lines had significantly longer roots (Fig. 3).

**Fig. 4:**
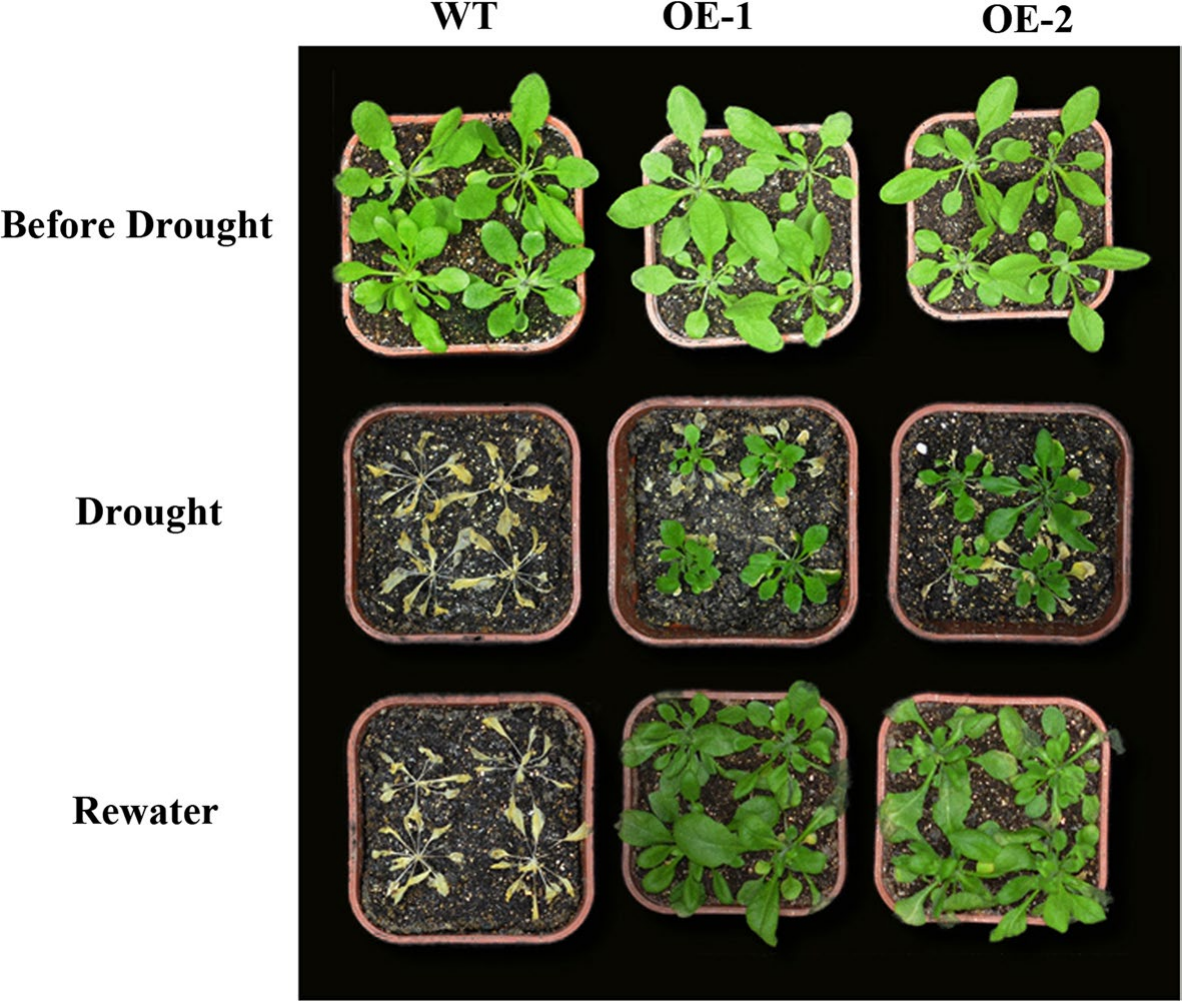
Analyse of drought tolerance in the adult stage. WT: wild-type plants; OE-1 and OE-2: transgenic lines. WT and transgenic plants were grown in soil with sufficient water for one month before water was withheld for 15 days, followed by recovery

**Fig. 5:**
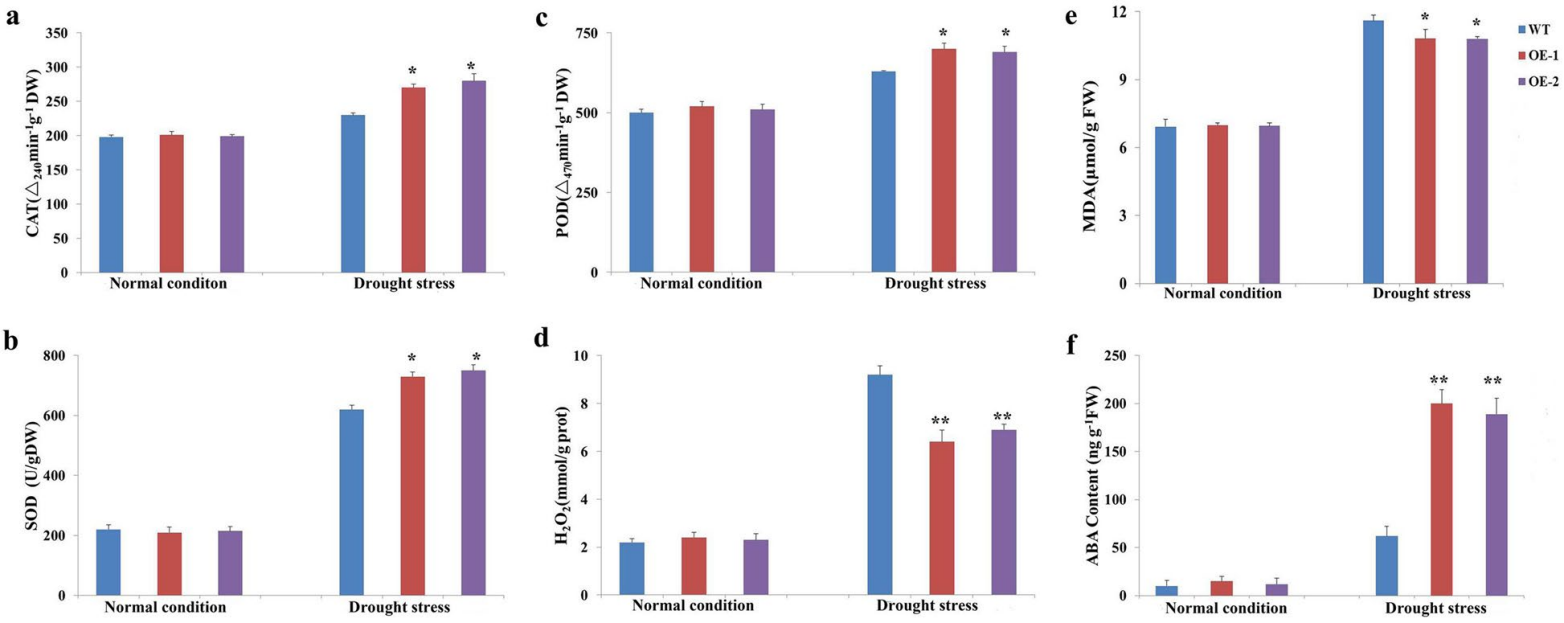
Detection of ROS scavenging capability, reactive oxygen species (ROS) accumulation, MDA and ABA concentrations under control and drought stress conditions. **a**-**c**: Activity levels of CAT, SOD and POD, respectively; (**d**): H_2_O_2_ concentration; (**e**): MDA concentration; (**f**): ABA content. WT: wild-type plants. OE-1 and OE-2: transgenic lines. Error bars represent SDs for three independent replicates. Asterisks indicate difference (*P < 0.05) and significant difference (**P < 0.01) comapring to WT and ()

In the adult stage (1 month old), no clear phenotypic difference was observed between the WT and transgenic lines in the absence of drought stress. When the plants were subjected to drought treatment, compared to the transgenic lines, the WT plants were more severely wilted (Fig. 4). To explore the mechanism through which *AhHLH112* overexpression improves drought stress resistance, the activities of the antioxidant enzymes catalase (CAT), peroxidase (POD), and superoxide dismutase (SOD) were measured after drought treatment. The activities of all three enzymes were significantly higher in the transgenic plants than in the WT plants. However, no significant differences were observed between the WT plants and transgenic plants under the CK conditions (Fig. 5a-c). Because the transgenic plants were more drought tolerant than the WT plants were, we measured the accumulation of hydrogen peroxide (H_2_O_2_). The level of H_2_O_2_ was lower in the overexpression plants than in the WT plants (Fig. 5d). We also measured the content of the drought-resistance indicator malondialdehyde (MDA) and ABA in the WT and transgenic plants. Under normal conditions, no differences in MDA and ABA content were found between the two types of plant. After drought treatment, the WT plants demonstrated higher MDA and lower ABA levels than the transgenic plants (Fig. 5e-f). Consistent with these results, detached leaves of transgenic plants lost water much more slowly than those of WT plants under drought stress (Fig. S7),

To further investigate the mechanisms underlying the improved drought tolerance achieved by overexpressing *AhHLH112*, the expression of the antioxidant genes *AtPOD* (AT5g66390), *AtCAT* (AT1G20630), and *AtSOD* (AT5G51100) in the WT and transgenic lines was measured via RT-qPCR. The transcription levels of antioxidant genes were higher in the transgenic lines than in the WT plants (Fig. S8) under drought condition, indicating that overexpression of *AhHLH112* led to upregulation of these genes. In addition, we measured the expression levels of two ABA-related genes under drought stress (*AtNCED3*: ABA-biosynthesis gene; *AtRD29A*: ABA stress-responsive gene). As shown in Fig. 7a, the expression level of *AtNCED3* among WT and transgenic lines were similar under normal condition. After drought treatment, the expression levels in two transgenic lines were higher than that of WT. The similar results were also found in *AtRD29A* (Fig. 7b) experiment. Under drought treatment, the expression level of *AtRD29A* in *AhHLH112*-overexpressing lines was significantly higher than this of WT plants. In summary, the results indicate that overexpression of *AhHLH112* may lead to improved drought tolerance in transgenic plants.

### AhbHLH112 directly binds to the promoter of AhPOD

**Fig. 6:**
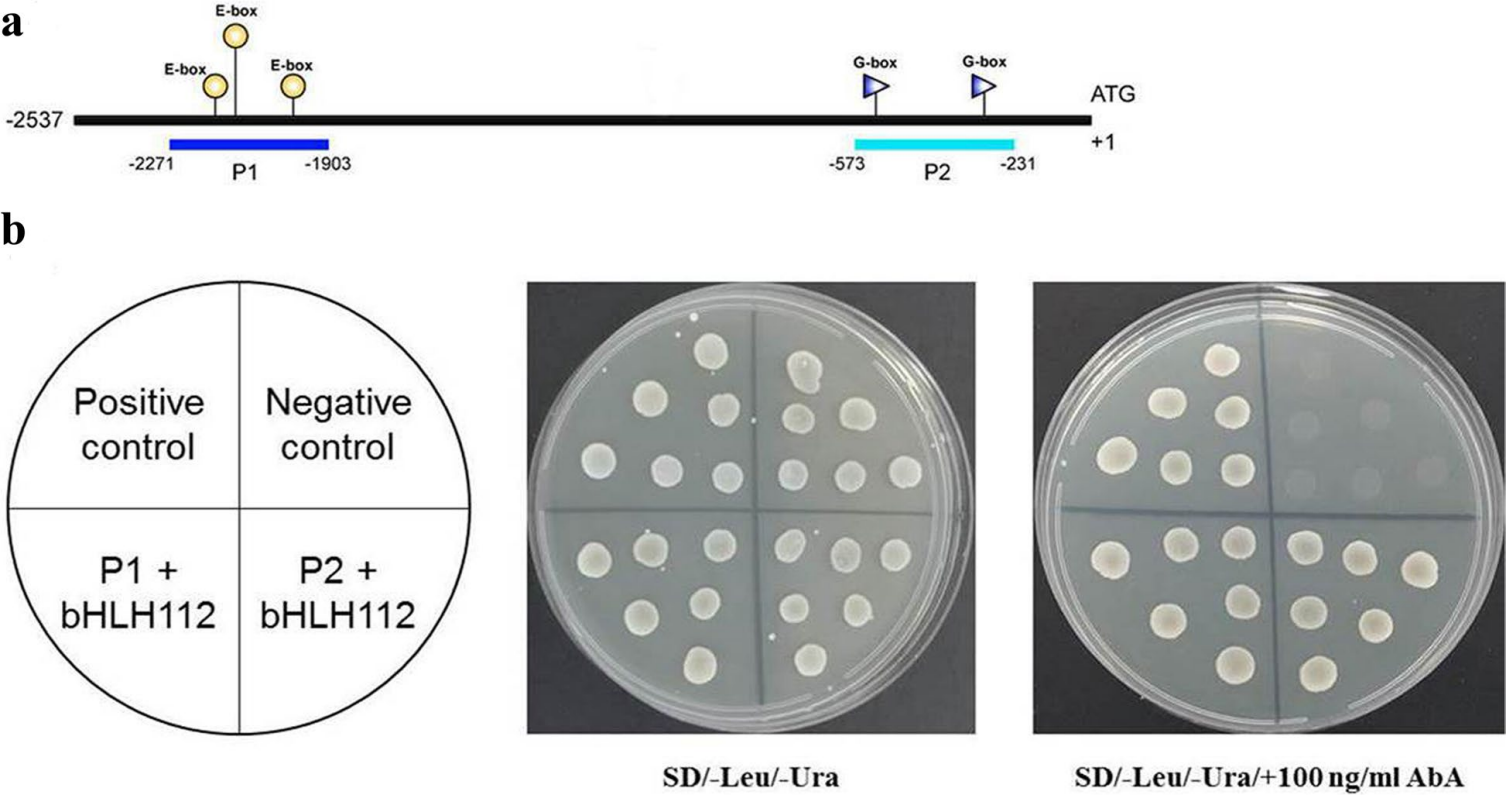
AhbHLH112 binds to and activates the promoter of *AhPOD*. **a**: Schematic diagrams of the promoters of *AhPOD*, in which the G-box elements are denoted using purple triangles, E-box elements are denoted using yellow circles. The segments marked with P1/2 represent the promoter fragments used in the yeast one-hybrid (Y1H) assay. **b**: Growth of yeast cells of positive control (p53-AbAi+pGAD-p53), negative control (bait+pGADT7), and co-transformants (bait+prey) on SD/−Leu/−Ura medium supplemented without (middle panel) or with (right panel) AbA

The *POD* gene was upregulated in the overexpression lines. We speculated that this gene may be regulated by *AhbHLH112*. To test our hypothesis, we obtained the promoter sequence of *AhPOD* (Arahy.IE3GQ3) and identified five G/E-box elements (Fig. 6a, Fig. S9). The interaction between AhbHLH112 and *AhPOD* promoter was investigated via Yeast one-hybrid (Y1H) assays. All of the yeast cells grew well on SD/−Leu/−Ura media, whereas the positive CK and yeast cells transformed with the effector and the P1 and P2 baits grew normally on media supplemented with 100 ng L^− 1^ aureobasidin A (AbA) (Fig. 6b). In brief, our results suggest that AhbHLH112 interacts with the P1 and P2 regions of the *AhPOD* promoter.

**Fig. 7:**
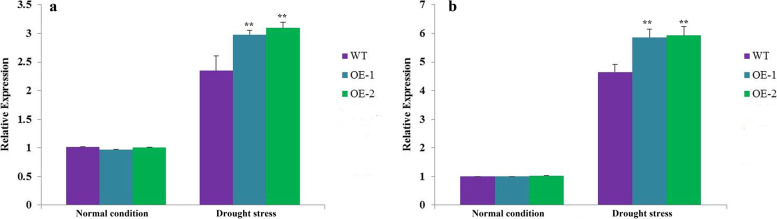
Relative expression levels of ABA-biosynthesis gene and ABA stress-responsive gene. Plants treated by normal condition and drought stress. a: Relative expression of *AtNCED3* (ABA-biosynthesis gene); b: Relative expression of *AtRD29A* (ABA stress-responsive gene). WT: wild-type plants. OE-1 and OE-2: transgenic lines. Data were presented as mean and SD values of three independent experiments. Asterisks indicated significant difference (** P < 0.01) comparing to WT.

## Discussion

The stress tolerance of a plant depends on adversity genes, and the overexpression of these genes can improve the plant’s ability to adapt to a variety of environmental stresses [28]. Although several studies of the involvement of bHLH TFs in plant abiotic stress response have been performed, including studies on drought stress [29–32], salt stress [33, 34], and chilling stress [35, 36], further studies are still required, particularly in the peanut [37, 38]. In-depth analysis and functional characterization of several of the family members can therefore provide an improved understanding of the members of the TF family in peanut involved in the response to stress. Here, we identified a TF, *AhHLH112*, with a potential role as a transcriptional activator regulating ROS-scavenging enzymes. Phylogenetic tree analyses indicated that the AhbHLH112 protein was clustered within the same clade as AtbHLH112 (At1g61660) of *Arabidopsis* and belonged to the bHLHs subgroup 12. *AtbHLH112* and its homologs from Oryza (*osbHLH68*) have been reported to be involved in abiotic stress and to control flowering [39, 40]. *AabHLH112* isolated from *Artemisia annua* is induced by low temperatures, and overexpression of *AabHLH112* significantly upregulates the expression levels of *AaERF1* and promotes artemisinin production [41]. From these studies, we conclude that the *bHLH112* of different plant species may exhibit diverse functions.

In this study, the expression of *AhHLH112* strongly increased in peanut plants exposed to drought, indicating that this gene is involved in abiotic stress responses. The expression patterns in the leaves, roots and stems under drought-stress conditions were different, suggesting that this gene is involved in different regulatory networks in different tissues. In addition, *AhHLH112* expression was more highly induced in the leaves than in other tissues, suggesting that this gene plays a more important role in the response to drought stress in leaves.

Our investigation also showed that WT *Arabidopsis* plants endows it with tolerance to drought stress, as revealed by the improved growth vigor of overexpressing plants under stress treatments in either the seedling or adult stage and the activities of antioxidant enzymes, POD, SOD, and CAT were significantly higher in transgenic plants than in the WT plants. In addition, the relative expression of correspondent genes was also higher in transgenic plants than in WT plants. It has been well established that antioxidant enzymes, which are regulated by TFs, play a predominant role in eliminating ROS accumulation under abiotic stresses [42]. ROS can cause oxidative damage to cellular components. Plant resistance to stress largely depends on the balance between ROS generation and scavenging [37, 43]. ROS-scavenging enzymes, such as POD, SOD, and CAT, are indispensable for ROS detoxification, with plants being able to maintain a better status under abiotic stress [43]. Other investigations were also verified that a number of genes could regulate the antioxidant enzymes to improve plant abiotic resistance. Magwanga [44] found that overexpression of *CYP450* gene in *Arabidopsis* could improve drought and salt tolerance level of the transgenic plants, and the antioxidant enzymes concentrations were significantly higher compared to the wild types under similar conditions. By promoting antioxidant enzymes concentrations, transcription factor *NtERF172* could confer drought resistance in tobacco (*Nicotiana tabacum*) [45]. Drought-induced transcription factor *XsWRKY20* was also identified as a positive regulator in drought stress through ROS homeostasis. Overexpression of *XsWRKY20* notably improved drought tolerance. Compared with the WT plants, the *XsWRKY20*-transgenic lines exhibited higher POD, CAT, SOD activities and lower ROS [46]. Herein, we hypothesize that by reducing ROS accumulation via modulation of antioxidant-scavenging machinery, *AhbHLH112* plays a role in the response of the peanut to drought stress.

Several studies have shown that at least 80 bHLHs have a specific amino acid composition in their DNA-binding domain required to bind to G-box elements [16, 37, 47, 48]. Many other bHLHs can bind to E-box elements, of which four nucleotides of the G-box core are retained (ACGT or CANNTG) [49]. For example, *AtbHLH122* has been reported to be a transcriptional activator that binds to G-box/E-box motifs to regulate gene expression [38, 39]. Furthermore, MYC2 is specifically associated with the E-box element of the *PLETHORA* gene promoter in *Arabidopsis* [50]. The expression of the antioxidant gene *AtPOD* was induced in the overexpression lines under drought conditions in the present research. This, in combination with the Y1H results, suggests that this gene may serve as a target of *AhHLH112* and that AhHLH112 protein interacts with the P1 and P2 regions (G/E-box) of the *AhPOD* promoter to control expression of the *POD* gene. Notably, SOD and CAT activities were also higher in the overexpression lines. However, more evidence is needed to verify whether the AhHLH112 protein can directly bind their promoters, which will be addressed in future work. In fact, the absence of an interaction between CsbHLH18 and the promoters of *CsSOD* and *CsCAT* has been observed, and CsbHLH18 binds only one E-box region of the *CsPOD* promoter [51]. Furthermore, Jiang et al. [52] showed that *RD29A*, *NCED3*, and *ABA3* were induced in response to overexpression of *AtWKRY57*, but only *RD29A* and *NCED3* are directly regulated by AtWRKY57.

As a critical plant hormone, ABA is involved in various developmental processes and stress-signaling transduction mechanisms in plants [53]. Several *bHLH* genes have been reported to induce ABA biosynthesis and to be involved in stress tolerance. For example, grape *VvbHLH1* confers great tolerance to drought stress in transgenic *Arabidopsis* by increasing ABA levels [54]. In agreement with this result, we also found the higher ABA content in transgenic lines than in WT under drought treatment, as well as the elevated expression levels of ABA-biosynthesis gene *AtNCED3* and ABA stress-responsive gene *AtRD29A*. These results suggested that *AhHLH112* might also positively function in plant defense via the ABA-dependent pathway.

## Conclusions

This study reported the characterization of *AhHLH112*, a bHLH transcription factor from peanut. Under drought stress, *AhHLH112* is induced and could activate antioxidant genes and promote ROS scavenging under drought stress. It also possibly participates in ABA-dependent stress-responding pathway. In addition, it acts upstream of *POD*, directly regulating its expression by binding to the G/E-box in the promoter region. Additional research is needed to determine whether *AhHLH112* directly regulates other antioxidant-related genes, such as *SOD* or *CAT*, to enhance drought tolerance. We conclude that *AhHLH112* exhibits important physiological functions in the drought stress response through the regulation of antioxidant gene-mediated ROS scavenging or ABA-dependent pathways, thus protecting plants against drought stress.

## Methods

### Experimental materials

The peanut cultivar HY9303 (developed by our group and registered by the Ministry of Agriculture and Rural Affairs, P.R. China) was used in this study. In our pre-experiment and investigation, HY9303 is a drought-resistance cultivar (not published). Seeds were planted at the Laixi Experimental Station of the Shandong Peanut Research Institute (36°51′00.00″N, 120°29′00.00″E) in Laixi, Shandong Province, China, with the permission of the Ministry of Agriculture and Rural Affairs, P.R. China (the certification of HY9303 is shown in Fig. S10). Complete uniformly growing seedlings were cultivated under the following conditions in temperature-controlled incubators (GXZ-260 C, Jiangnan, China): fertilizer, Hoagland’s culture medium; humidity, 60%; photoperiod, 16 h/8 h (day/night); temperature, 26 °C during the day (with 100 μmol m^− 2^ s^− 1^ irradiance) and 22 °C during the night. The seedlings were cultivated for 21 days.

### Sequence and expression analysis of *AhbHLH112*

As mentioned above, a *bHLH* gene was found to be one of the most drought-inducible TFs in peanut [1]. By blasting the whole-genome of peanut (www.peanutbase.org), we identified it as *AhbHLH112* (Arahy.0I3NZ2). Total RNA was isolated from the roots of 21-day-old greenhouse-grown plants using Takara MiniBEST Plant RNA Extraction Kit (Takara, Dalian, China) and reverse transcribed into cDNA using a PrimeScript™ II 1st Strand cDNA Synthesis Kit (Takara, Dalian, China). Using the whole-genome sequence of cultivated peanut (www.peanutbase.org), we isolated *AhbHLH112* cDNA with PrimeSTAR® GXL DNA Polymerase (Takara). All of the primer pairs used are listed in Table S1. The amplified products were inserted into *pEASY*®-Blunt Simple Cloning Vectors (TransGen, Beijing, China) and verified by sequencing. The molecular mass and theoretical isoelectric point of the protein were predicted with DNAMAN 6.0. For conserved domain searches, we used the NCBI database (https://www.ncbi.nlm.nih.gov/Structure/cdd/wrpsb.cgi?). Using the manually aligned bHLH region of 142 bHLH proteins from *Arabidopsis* [55] and AhbHLH112 protein, a phylogenetic tree was constructed using the neighbor-joining method with MEGA 6.0. Bootstrapping was done with 1000 replicates to assess the statistical reliability of the nodes in the tree.

The seedlings of HY9303 were cultivated for 21 days in temperature-controlled incubators (GXZ-260 C, Jiangnan, China). Then, the 21-one-day-old greenhouse-grown plants were used as samples. The roots, stems, and leaves were separately collected from the control (CK) samples under normal conditions. Drought stress was created by supplementing Hoagland’s solution with 20% polyethylene glycol (PEG) 6000 [56, 57]. The roots, stems, and leaves were collected at 6, 12, 18, 24, and 48 h post-stress (hps) before being frozen immediately in liquid nitrogen (constituting the experimental groups) and were stored at − 80 °C until use. Expression profiles of *AhbHLH112* were analyzed via RT-qPCR. The primers used for RT-qPCR were designed using Beacon Designer 7.0. The expression levels of the selected genes were normalized against those of *Actin 11*, which was used as an internal CK. The reactions were performed according to the SYBR Premix Ex Taq™ protocol using an Applied Biosystems 7500 Fast Real-Time PCR System (ABI, USA), with a 20 μL reaction mixture, following the manufacturer’s recommendations. Three biological replicates were included for the selected genes, and the relative gene-expression levels were calculated using the 2^−ΔΔCT^ method. All of the primers used are listed in Table S1.

### Subcellular localization and transcriptional activation

Subcellular localization was determined as described in previous research [58]. We cloned *AhbHLH112* into a pCAMBIA2300-GFP donor vector at the XbaI and SalI restriction sites and generated a pCAMBIA2300-AhbHLH112-GFP plasmid. pCAMBIA2300-GFP was used as the CK. The plasmids were transiently transformed into *Nicotiana tabacum* leaves by *Agrobacterium tumefaciens* infiltration. The infiltrated plants were cultivated in the dark for 8 h and then grown for an additional 2 days under a 16 h light/8 h darkness photoperiod. A laser scanning confocal microscope (SP8, Leica, Germany) with an excitation wavelength of 488 nm was used for observations.

The coding sequence of *AhbHLH112* was subcloned into a pGBKT7 vector (Clontech), yielding pGBKT7-AhbHLH112. The vector constructs were subsequently transformed into yeast Y2H Gold competent cells using the lithium acetate method (PT1172–1, Clontech, Japan). The transformed yeast cells were grown on selection media that included SD/−Trp and SD/−Trp/−His/−Ade at 30 °C in the dark for 3 days and then incubated together with 20 μg mL^− 1^ X-α-gal to form blue clones. pGBKT7–53 was used as a positive CK. The growth status of the yeast colonies and β-galactosidase activity were used to identify transcriptional activity.

### Yeast one-hybrid (Y1H) assays

The promoter of *AhPOD* (Arahy.IE3GQ3) was acquired via genomic PCR with the specific primers shown in Table S1, using peanut genomic DNA used as a template. Two promoter fragments (P1/2 for *AhPOD*), which included the G/E-box, were amplified and ligated into a pAbAi vector as bait. The full-length *AhbHLH11*2 CDS was amplified and fused to the pGADT7 vector as prey. Y1H assays were performed via a Matchmaker Gold Yeast One-Hybrid Library Screening System (Clontech, USA) following the manufacturer’s protocol.

### Transformation and characterization of transgenic plants

We cloned the CDS of *AhbHLH112* into a pCAMBIA2300 vector at the XbaI and SalI restriction sites, yielding a pCAMBIA2300-AhbHLH112 overexpression vector. *Arabidopsis* plants were transformed as previously reported [59]. The transformants were selected on 1/2-strength Murashige and Skoog (MS) media including 50 μg mL^− 1^ kanamycin. The T_3_ homozygous lines were used for analyses. The presence of the transgene was confirmed via genomic PCR, and the expression levels of the transgene were measured via RT-PCR.

For drought stress assays at the seedling stage, sterilized seeds from each type of *Arabidopsis* plant (transgenic and CK plants) were placed on 1/2-strength MS solid media, supplemented with 250 mM mannitol. The culture dishes were placed vertically and incubated under a 16 h/8 h (day/night) photoperiod at 20 °C, an irradiance of 100 μmol m^− 2^ s, and 65% relative humidity. The taproot length of each sample (10 seedlings per line per petri dish) was measured 10 days later.

To explore the drought tolerance of mature plants, the seeds of each line were sown in pots and subsequently grown under regular cultivation conditions, and 1-month-old plants were subjected to drought stress. For drought treatment, the watering was stopped immediately and continued for 15 days, and then rewatered and imaged [31, 60]. SOD, POD, and CAT activities and MDA contents were determined as described previously [61, 62], ABA was quantified using a high-performance liquid chromatography (HPLC)-electrospray ionization-tandem mass spectrometry method by comparing the peak areas with those of known amounts of standard ABA [63]. H_2_O_2_ was measured with a hydrogen peroxide assay kit (Jiancheng Bioengineering Institute, Nanjing, China). Water loss was represented as the percentage of initial fresh weight as described by Liu [38]. Each experiment was performed with three biological replicates. The expression levels of antioxidant genes and ABA response genes were normalized against those of *UBC*, which was used as an internal CK [58]. The reactions were performed as mentioned above. Three biological replicates were included for the selected genes, and the relative gene-expression levels were calculated using the 2^−ΔΔCT^ method. All of the primers used are listed in Table S1.

## Supplementary Information

**Additional file 1: Table S1**: Primers used in this study

**Additional file 2: Figure S1**. Comparison the sequences of *AhbHLH11*2 from three sources. AH10G04090: gene ID of *AhbHLH11*2 which was downloaded from http://peanutgr.fafu.edu.cn/index.php; Arahy.0I3NZ2.1: gene ID of *AhbHLH11*2 which was downloaded from peanutbase, www.peanutbase.org; AhbHLH112: sequence obtained in this study.

**Additional file 3: Figure S2**. Analyses of full sequence of *AhbHLH112*. Red: exon of *AhbHLH112*. Yellow: intron of *AhbHLH112*.

**Additional file 4: Figure S3**. Conserved domain analyses of AhbHLH112 protein

**Additional file 5: Figure S4**. Phylogenetic analyses of AhbHLH112 protein based on bHLH domains from *Arabidopsis thaliana.* Arrow was pointed to AhbHLH112. At1g61660: gene ID of *AtbHLH112* in *Arabidopsis thaliana.*

**Additional file 6: Figure S5**: Selection of transformants by PCR. M: DL2000 (Takara, Dalian, China); WT: wild-type *Arabidopsis*; OE-1 and OE-2: the two lines of transgenic *Arabidopsis*. Marker size (from up to down): 2000 bp, 1000 bp, 750 bp, 500 bp, 200 bp, and 100 bp. The band size of target gene was about 1300 bp.

**Additional file 7: Figure S6**. The transcript level of *AhbHLH112* overexpressing in transgenic *Arabidopsis* and wild type plants assayed by RT-PCR. WT: wild-type *Arabidopsis*; OE-1 and OE-2: the two lines of transgenic *Arabidopsis*. M: DL2000 (Takara, Dalian, China); UBC (AT5g25760) was used as an internal control. The expression of UBC in WT, OE-1 and OE-2 is shown in the left of M. UBC was expressed in WT, OE-1 and OE-2. Expression of *AhbHLH112* in WT, OE-1 and OE-2 is shown to the right of M. *AhbHLH112* was expressed in OE-1 and OE-2 Marker size (from up to down): 2000 bp, 1000 bp, 750 bp, 500 bp, 200 bp, and 100 bp. The band size of target gene was about 1300 bp.

**Additional file 8: Figure S7**. Water loss from detached leaves of WT and two transgenic plants under drought stress. WT: wild-type *Arabidopsis*; OE-1 and OE-2: the two lines of transgenic *Arabidopsis*.Water loss was represented as the percentage of initial fresh weight. Data are presented as means and SDs of three independent experiments. Asterisks indicate significant difference (**P < 0.01) comparing to WT.

**Additional file 9: Figure S8**. Gene expression level of antioxidant enzyme (*AtCAT*, *AtPOD*, and *AtSOD*) in the transgenic lines and wild-type plants under normal and drought stress conditions. WT: wild-type *Arabidopsis*; OE-1 and OE-2: the two lines of transgenic *Arabidopsis*. (a): Gene-expression level of *AtCAT* (AT1G20630); (b): Gene-expression level of *AtPOD* (AT5g66390)*;* (c): Gene-expression level of *AtSOD* (AT5G51100). UBC (AT5g25760) was used as an internal reference control, and the transcript level of the tested gene was calculated using the 2 ^−∆∆CT^ method. Error bars represent SDs for three independent replicates. Asterisks ** indicate a significant difference comparing to WT (*P* < 0.01).

**Additional file 10: Figure S9**. G/E-box analyses of promoter of *AhPOD*. Yellow: E-box; Green: G-box

**Additional file 11: Figure S10**. Cultivar registration certificate of ‘HY9303’ (in Chinese)

## Data Availability

The datasets used and analyzed in the current study are available from the corresponding author on reasonable request.

## Abbreviations

TFs: Transcription factors
bHLH: Basic helix-loop-helix
ROS: Reactive oxygen species
ABA: Abscisic acid
WT: Wild type
CAT: Catalase
POD: Peroxidase
SOD: Superoxide dismutase
MDA: Malondialdehyde
Y1H: Yeast one-hybrid
AbA: Aureobasidin A
CDS: Coding DNA sequence
PEG: Polyethylene glycol
MS: Murashige and Skoog
H_2_O_2_: Hydrogen peroxide
JS: Jensen-Shannon
