# Supplementary material for: The bHLH transcription factor AhbHLH112 improves the drought tolerance of peanut
Source: BMC Plant Biol. 2021 Nov 16;21:540. doi: 10.1186/s12870-021-03318-6 (PMC8594184; doi:10.1186/s12870-021-03318-6)
Supplement: Supplementary file 1 — Additional file 1: Table S1: Primers used in this study [file 12870_2021_3318_MOESM1_ESM.docx]

Table S1 Primers used in this study

| Primers | Sequences(5'-3') | Goal |
| --- | --- | --- |
| 112-FL-F | TTTGGTAGTTGCCTCCAACC | Clone for full length of *AhbHLH112* |
| 112-FL-R | CCACATGCTAAATTCAAGGCAC |  |
| 112-CDS-F | ATGGCTGAGGAGTTTCAAGCTACAA | Clone for CDS of *AhbHLH112* |
| 112-CDS-R | TTGATGACTACCTGCCACTGCCA |  |
| 2300-AhbHLH112-F | AGAACACGGGGGACGAGCTCATGGCTGAGGAGTTTCAAGCTACAA | Subcellular localization |
| 2300-ArbHLH112-R | ACCATGGTGTCGACTCTAGATTGATGACTACCTGCCACTGCCA |  |
| Vigs-112-f | GGCCTCGAGACGCGTGAGCTCTATGACTCTTTCTGGCAAGG | Amplified for VIGS system |
| Vigs-112-r | GTGAGTAAGGTTACCGAATTCGTGACAGTTCAGGAAGGGT |  |
| Actin11-f | TTGGAATGGGTCAGAAGGATGC | Actin of RT-qPCR in peanut |
| Actin11-r | AGTGGTGCCTCAGTAAGAAGC |  |
| R-112-f | TCCATCGCCATATTATTC | RT-qPCR of *AhbHLH112* in peanut |
| R-112-r | TTATGAGATTGAAGGTGTT |  |
| UBC (AT5g25760) F | CTGCGACTCAGGGAATCTTCTAA | Actin of RT-qPCR in Arabidopsis |
| UBC (AT5g25760) R | TTGTGCCATTGAATTGAACCC |  |
| Pro-AhPOD-f | GAATATGTACTTTATGAAAACCAATTTTC | Amplified for promoter of *AhPOD* |
| Pro-AhPOD-r | GCTGGCAATGAGGGAAATTATCC |  |
| At-POD-f(AT5g66390) | TATGGAAGTGGCGGCTAT | Amplified of *AtPOD* for RT-qPCR |
| At-POD-r(AT5g66390) | ACAATGGACTGAACAATCTCTT |  |
| At-CAT-f(AT1G20630) | TATTCTTCGTCCGTGATG | Amplified of *AtCAT* for RT-qPCR |
| At-CAT-r(AT1G20630) | CAGTATCCTCCAGTTCTC |  |
| At-SOD-f(AT5G51100) | CATATAAGGCGAATAGAC | Amplified of *AtSOD* for RT-qPCR |
| At-SOD-r AT5G51100) | GAGAAGTGGAGAATAATC |  |
| AD_b112-F | ATGGCCATGGAGGCCAGTGAATTCATGGCTGAGGAGTTTCAAGCTAC | For Y1H test |
| AD_b112-R | CTGCAGCTCGAGCTCGATGGATCCTCATTGATGACTACCTGCCACTG |  |
| POD_P1-F | ATTGAAAAGCTTGAATTCGAGCTCGGCTGTTAACAGTGTTCCATATTACG |  |
| POD_P1-R | TACATACAGAGCACATGCCTCGAGCTGACATGTTCAATCTTGACTGTG |  |
| POD_P2-F | ATTGAAAAGCTTGAATTCGAGCTCGAACCCAAGGACTGAATTGTTCACC |  |
| POD_P2-R | TACATACAGAGCACATGCCTCGAGGTGTTGACTTGTTAACCATCCTCC |  |
