# Supplementary figures and images for: The bHLH transcription factor AhbHLH112 improves the drought tolerance of peanut
Source: BMC Plant Biol. 2021 Nov 16;21:540. doi: 10.1186/s12870-021-03318-6 (PMC8594184; doi:10.1186/s12870-021-03318-6)

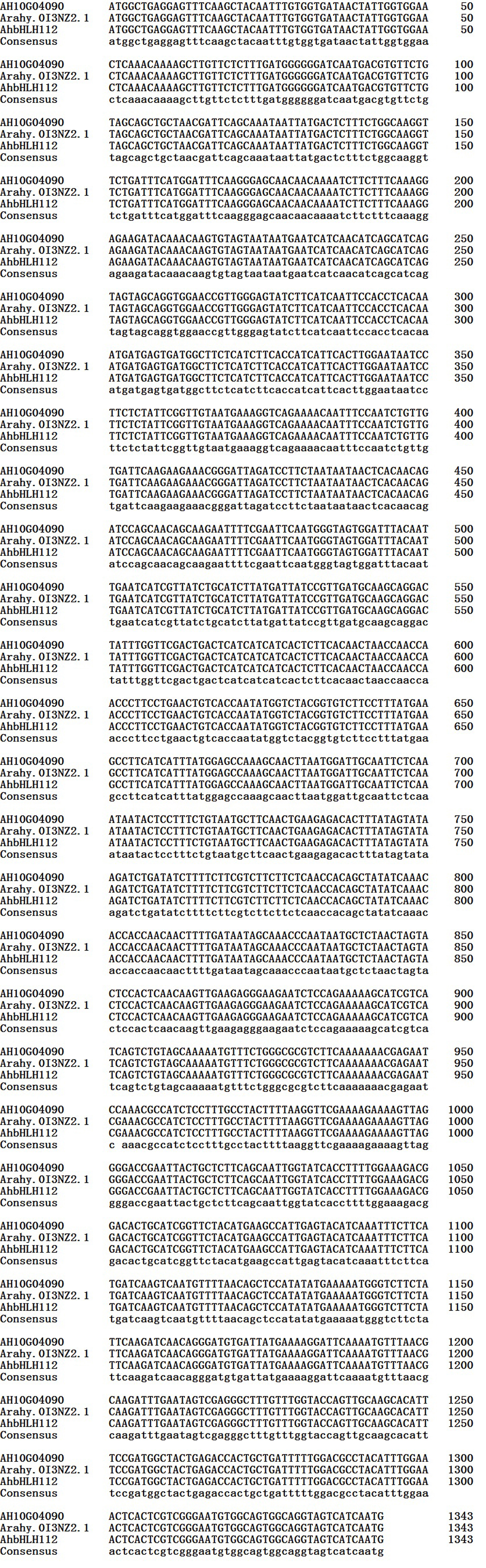

Supplement: Supplementary file 2 — Additional file 2: Figure S1. Comparison the sequences of AhbHLH112 from three sources. AH10G04090: gene ID of AhbHLH112 which was downloaded from http://peanutgr.fafu.edu.cn/index.php; Arahy.0I3NZ2.1: gene ID of AhbHLH112 which was downloaded from peanutbase, www.peanutbase.org; AhbHLH112: sequence obtained in this study. [file 12870_2021_3318_MOESM2_ESM.jpg]

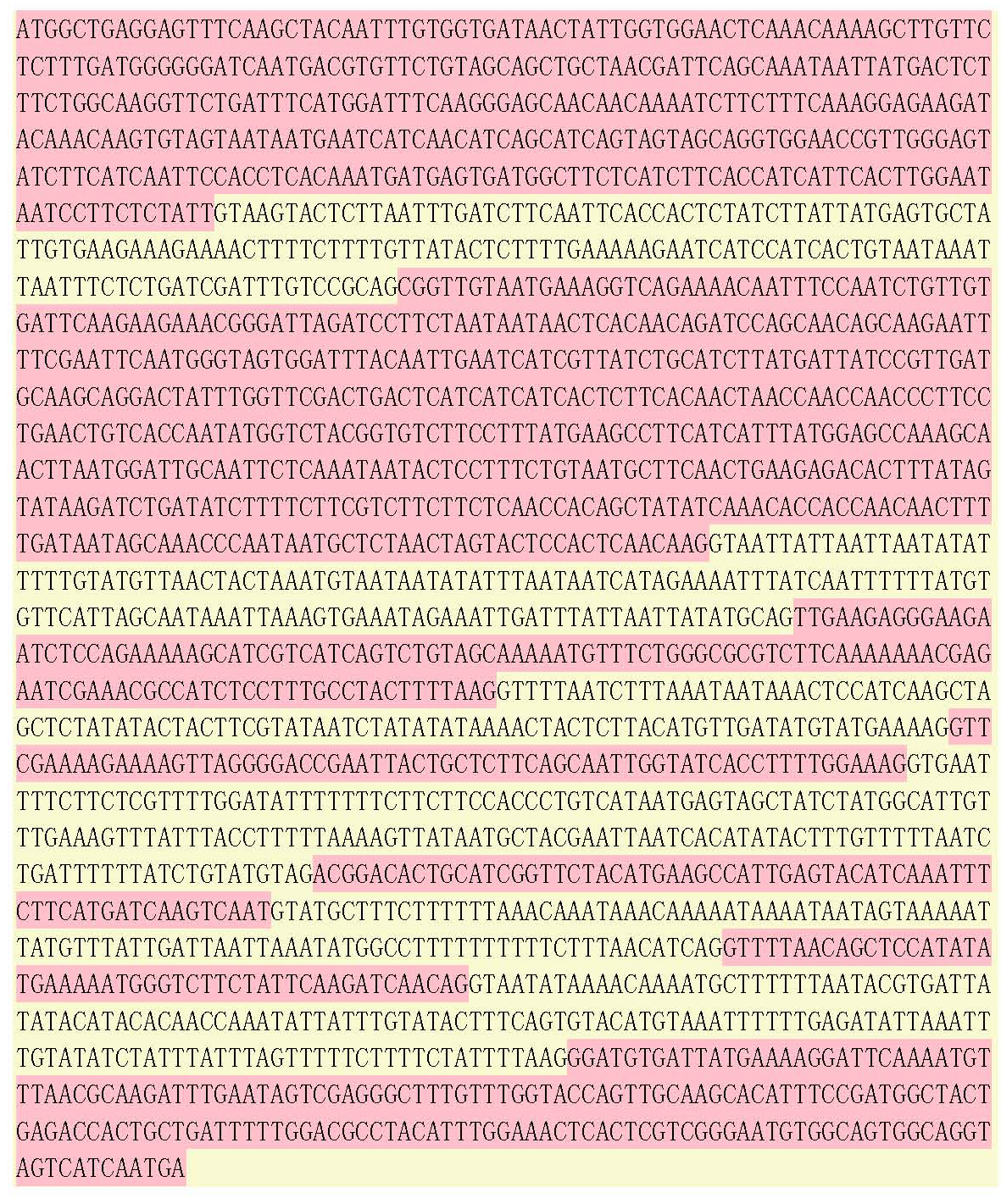

Supplement: Supplementary file 3 — Additional file 3: Figure S2. Analyses of full sequence of AhbHLH112. Red: exon of AhbHLH112. Yellow: intron of AhbHLH112. [file 12870_2021_3318_MOESM3_ESM.jpg]

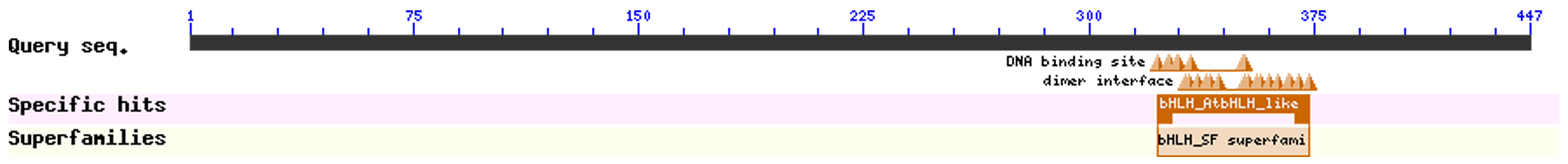

Supplement: Supplementary file 4 — Additional file 4: Figure S3. Conserved domain analyses of AhbHLH112 protein [file 12870_2021_3318_MOESM4_ESM.jpg]

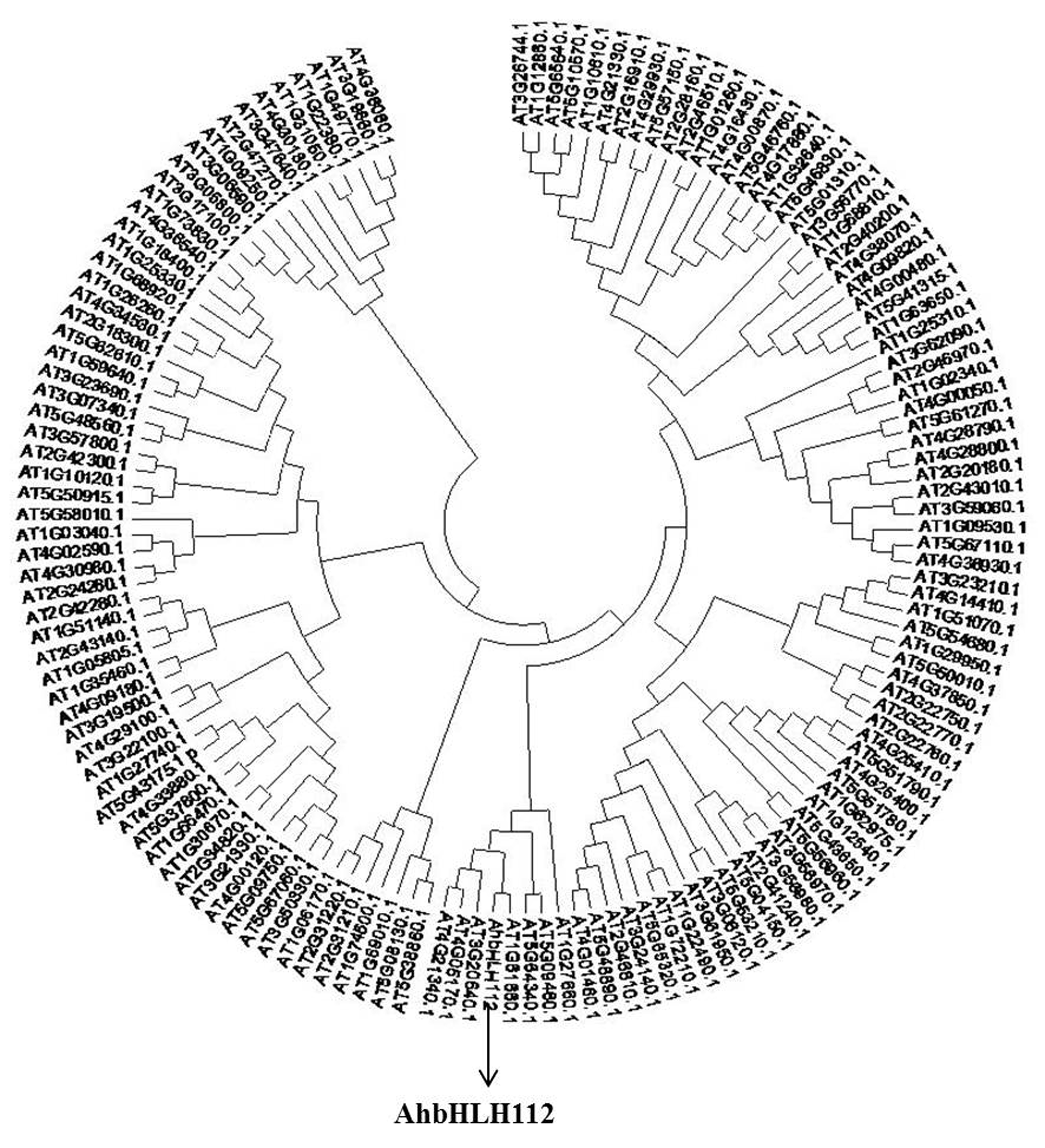

Supplement: Supplementary file 5 — Additional file 5: Figure S4. Phylogenetic analyses of AhbHLH112 protein based on bHLH domains from Arabidopsis thaliana. Arrow was pointed to AhbHLH112. At1g61660: gene ID of AtbHLH112 in Arabidopsis thaliana. [file 12870_2021_3318_MOESM5_ESM.jpg]

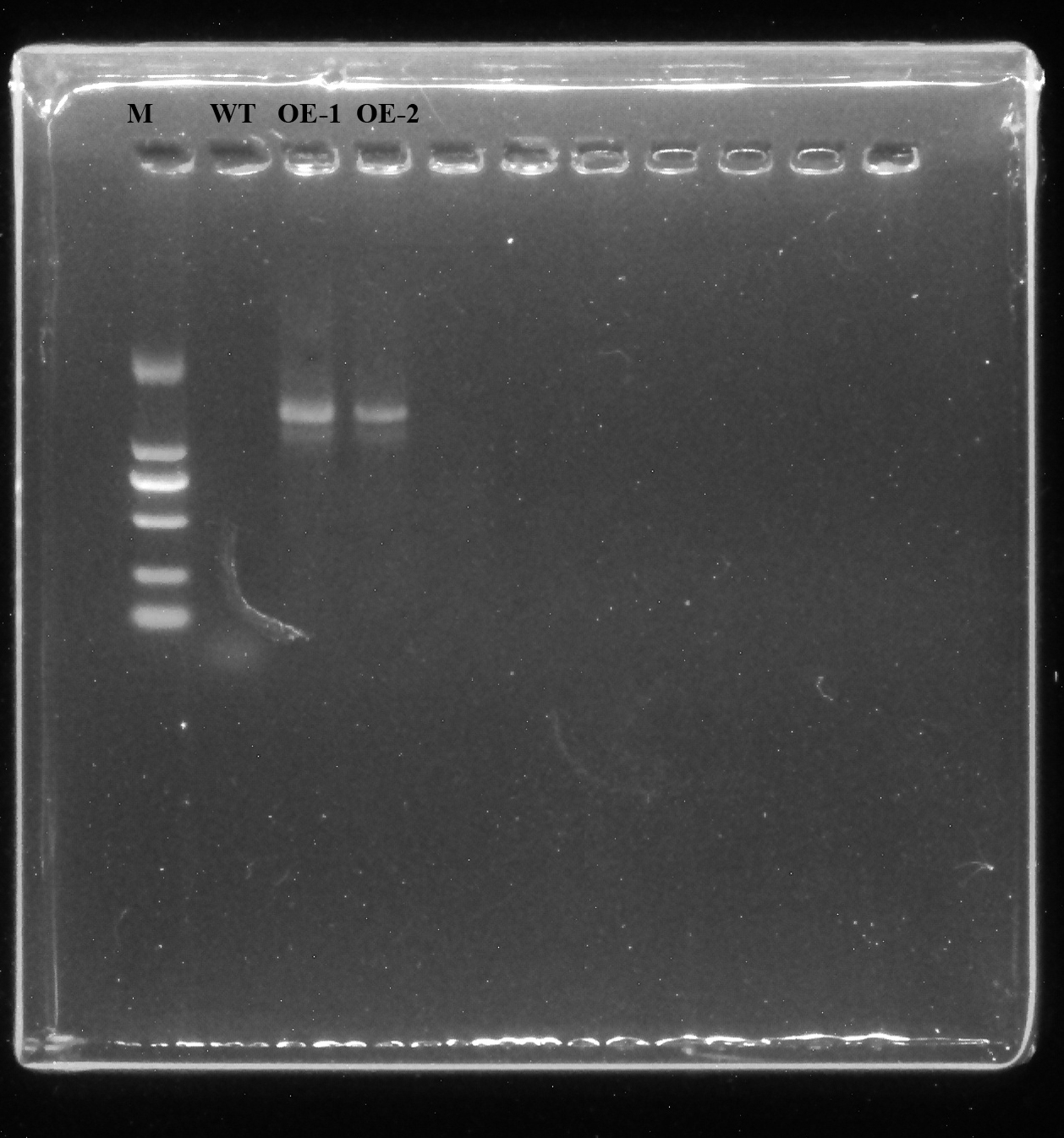

Supplement: Supplementary file 6 — Additional file 6: Figure S5: Selection of transformants by PCR. M: DL2000 (Takara, Dalian, China); WT: wild-type Arabidopsis; OE-1 and OE-2: the two lines of transgenic Arabidopsis. Marker size (from up to down): 2000 bp, 1000 bp, 750 bp, 500 bp, 200 bp, and 100 bp. The band size of target gene was about 1300 bp. [file 12870_2021_3318_MOESM6_ESM.tif]

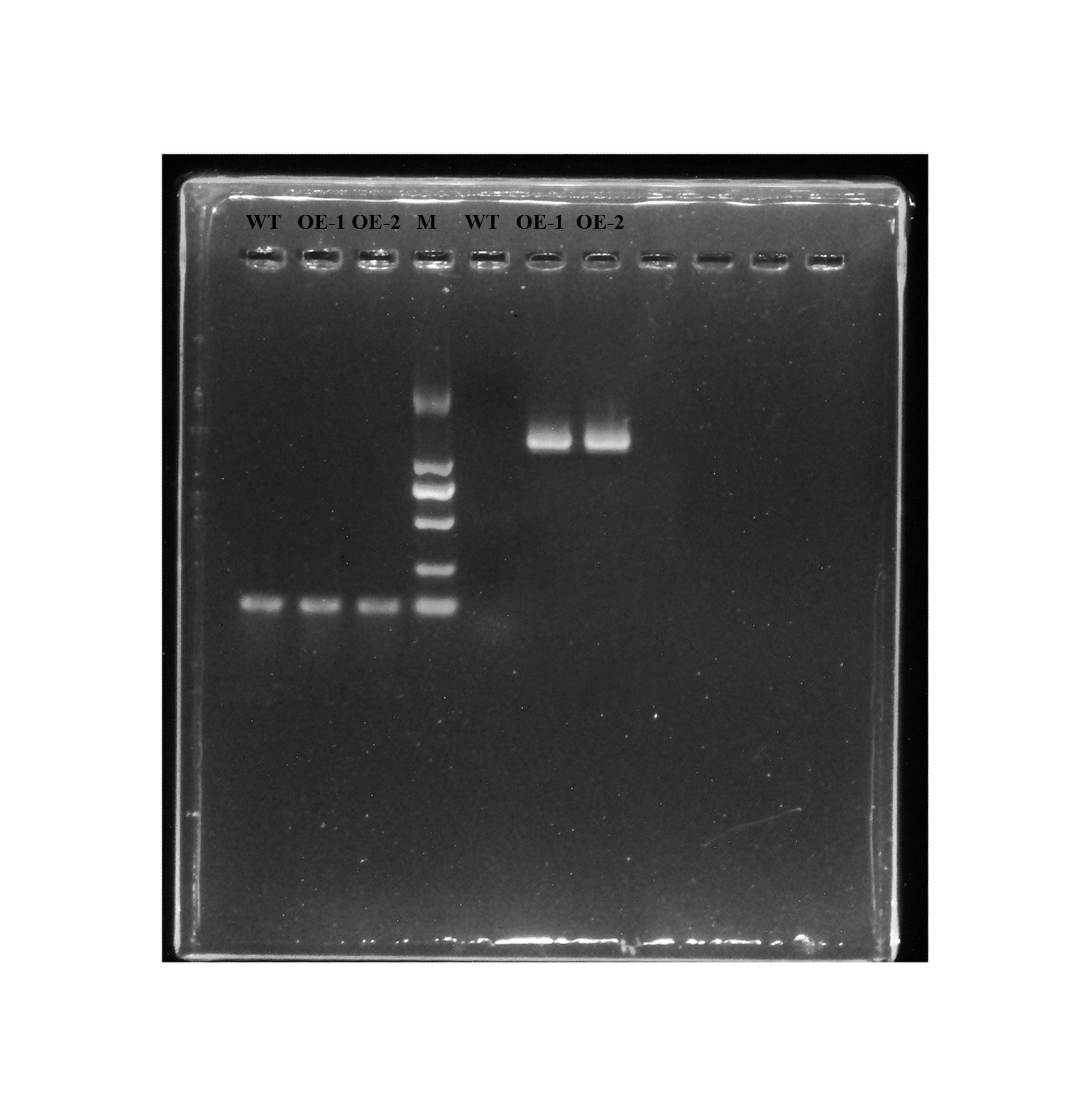

Supplement: Supplementary file 7 — Additional file 7: Figure S6. The transcript level of AhbHLH112 overexpressing in transgenic Arabidopsis and wild type plants assayed by RT-PCR. WT: wild-type Arabidopsis; OE-1 and OE-2: the two lines of transgenic Arabidopsis. M: DL2000 (Takara, Dalian, China); UBC (AT5g25760) was used as an internal control. The expression of UBC in WT, OE-1 and OE-2 is shown in the left of M. UBC was expressed in WT, OE-1 and OE-2. Expression of AhbHLH112 in WT, OE-1 and OE-2 is shown to the right of M. AhbHLH112 was expressed in OE-1 and OE-2 Marker size (from up to down): 2000 bp, 1000 bp, 750 bp, 500 bp, 200 bp, and 100 bp. The band size of target gene was about 1300 bp. [file 12870_2021_3318_MOESM7_ESM.tif]

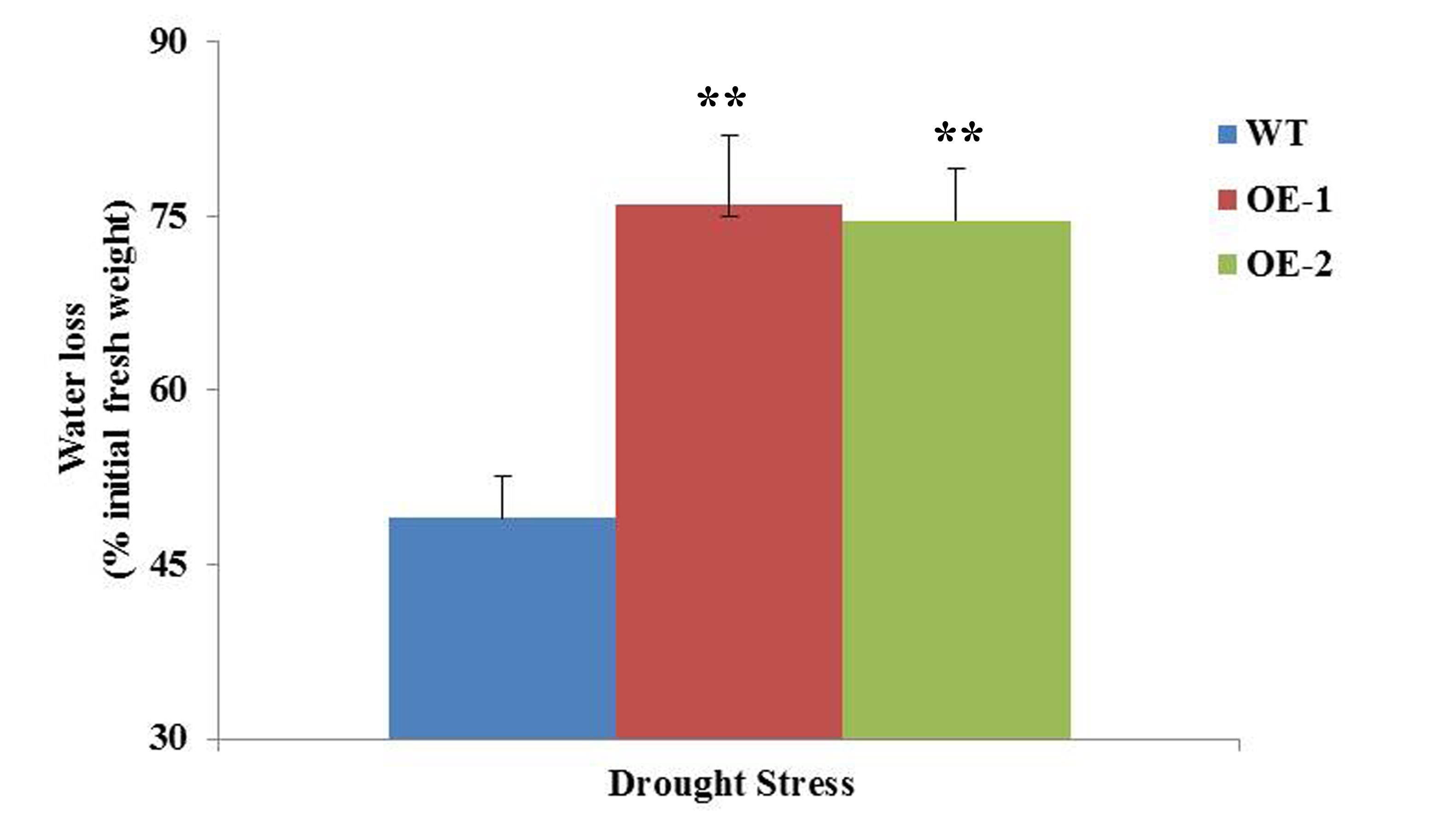

Supplement: Supplementary file 8 — Additional file 8: Figure S7. Water loss from detached leaves of WT and two transgenic plants under drought stress. WT: wild-type Arabidopsis; OE-1 and OE-2: the two lines of transgenic Arabidopsis.Water loss was represented as the percentage of initial fresh weight. Data are presented as means and SDs of three independent experiments. Asterisks indicate significant difference (**P < 0.01) comparing to WT. [file 12870_2021_3318_MOESM8_ESM.jpg]

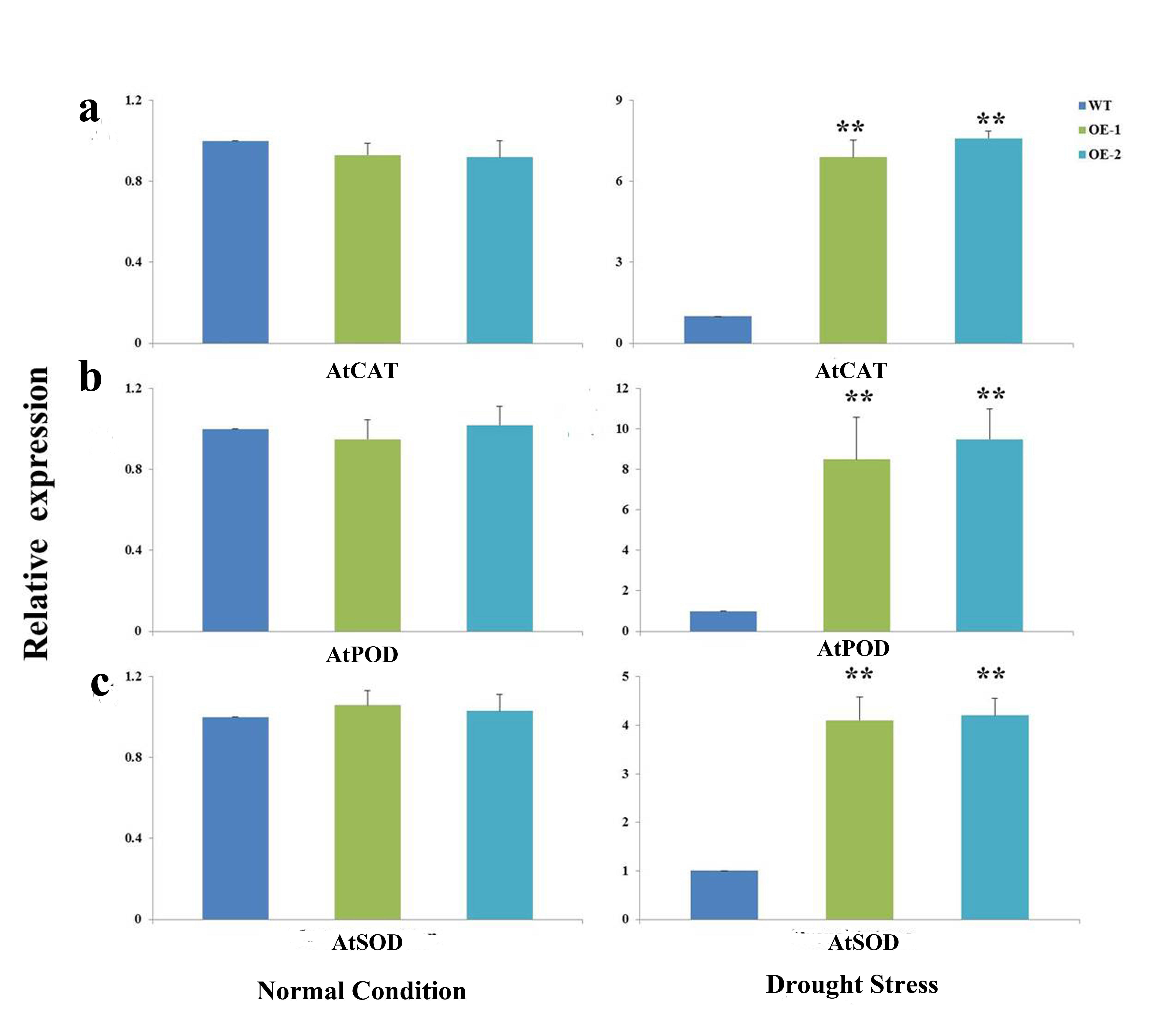

Supplement: Supplementary file 9 — Additional file 9: Figure S8. Gene expression level of antioxidant enzyme (AtCAT, AtPOD, and AtSOD) in the transgenic lines and wild-type plants under normal and drought stress conditions. WT: wild-type Arabidopsis; OE-1 and OE-2: the two lines of transgenic Arabidopsis. (a): Gene-expression level of AtCAT (AT1G20630); (b): Gene-expression level of AtPOD (AT5g66390); (c): Gene-expression level of AtSOD (AT5G51100). UBC (AT5g25760) was used as an internal reference control, and the transcript level of the tested gene was calculated using the 2 −∆∆CT method. Error bars represent SDs for three independent replicates. Asterisks ** indicate a significant difference comparing to WT (P < 0.01). [file 12870_2021_3318_MOESM9_ESM.jpg]

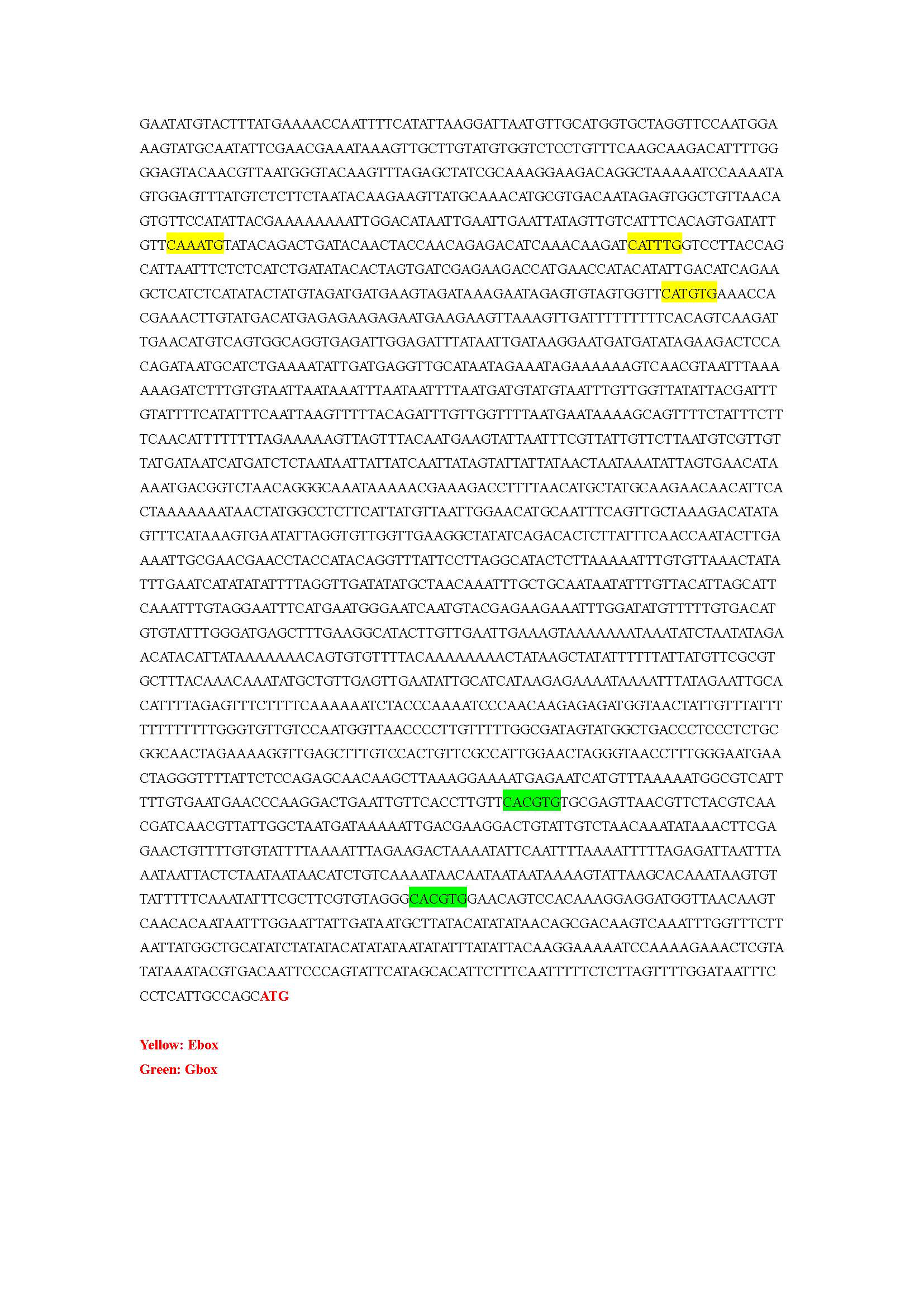

Supplement: Supplementary file 10 — Additional file 10: Figure S9. G/E-box analyses of promoter of AhPOD. Yellow: E-box; Green: G-box [file 12870_2021_3318_MOESM10_ESM.jpg]

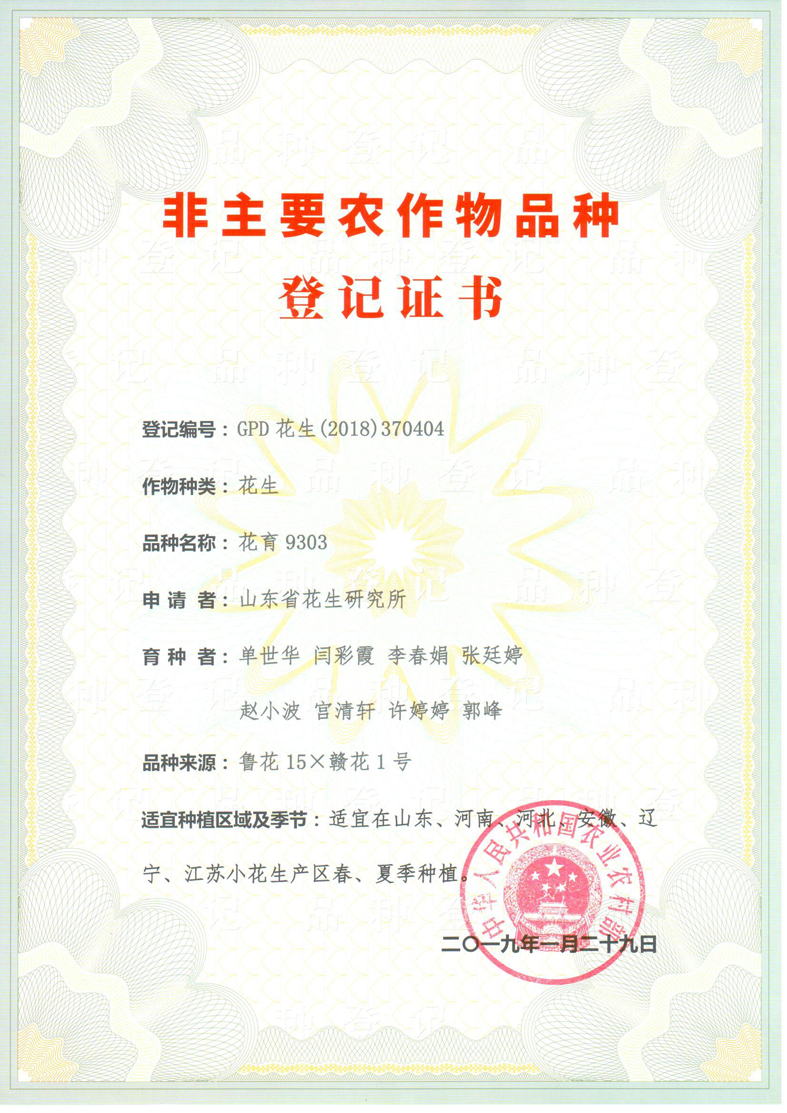

Supplement: Supplementary file 11 — Additional file 11: Figure S10. Cultivar registration certificate of ‘HY9303’ (in Chinese) [file 12870_2021_3318_MOESM11_ESM.tif]
